# Relationship between Interferon Beta-1A Administration and Intracranial Vascular Tone Regulation in Patients with Relapsing-Remitting Multiple Sclerosis: A Pilot Study

**DOI:** 10.1155/2017/5421416

**Published:** 2017-09-13

**Authors:** Vincenzo Dattola, Lilla Bonanno, Antonino Naro, Antonino Chillura, Anna Lisa Logiudice, Edoardo Sessa, Fausto Famà, Angelo Quartarone, Rocco Salvatore Calabrò, Silvia Marino, Margherita Russo

**Affiliations:** ^1^IRCCS Centro Neurolesi “Bonino Pulejo”, Via P.le Palermo S.S. 113, 98124 Messina, Italy; ^2^Department of Human Pathology, University Hospital of Messina, Via Consolare Valeria, Messina, Italy; ^3^Department of Clinical and Experimental Medicine, University Hospital of Messina, Via Consolare Valeria, Messina, Italy

## Abstract

Interferon beta (IFN-*β*) therapy is one of the most commonly prescribed immunomodulatory therapies in relapsing-remitting multiple sclerosis (RRMS). A reversible cerebral vasoconstriction syndrome (RCVS), associated with IFN-*β* use, has been recently described. For this reason, we tested the effect of once a week intramuscular administration of IFN-*β*-1A on the function of cerebral vessels in a cohort of RRMS patients. Using transcranial Doppler (TCD) ultrasound, we measured the mean blood flow velocity (MFV) in intracranial vessels 10 h after IFN-*β* administration. Measurements showed a significant increase in MFV compared to the baseline values in some vessels.

## 1. Background

Multiple sclerosis (MS) is a chronic, autoimmune, and predominantly demyelinating disease involving the central nervous system (CNS) [1]. The disease affects mainly young females (female/male ratio about 2 : 1), between the second and fourth decade of life, and represents the second cause of neurological disability, after traumatic brain injury [2].

To date, due to its complex etiopathogenesis, probably resulting from genetic and environmental factors (only partially known) interaction, no curative treatment is available for MS [1, 2].

However, over the past 30 years, various drugs have been introduced into clinical practice, namely, disease modifying therapies (DMTS), able to modify the clinical course of the disease [3].

The interferon beta (IFN-*β*), 1A and 1B, represents the first widely used DMTS, and it is still one of the most commonly administered drugs for the treatment of the relapsing-remitting MS (RRMS) variant [4].

IFNs are a group of endogenous glycoproteins endowed with immunomodulatory, antiviral, and antiproliferative properties [5]. The mechanism of action lays in their ability to modulate immune system activity mainly by reducing the migration of peripheral T-lymphocytes to the CNS [5, 6]. Side effects are widely known and there are also many complications related to chronic IFN treatment. The most common is represented by the flu-like syndrome, whose symptoms (e.g., headache, fever, and ubiquitary arthralgia) are prominent at the beginning of the therapy, although they decrease progressively, in frequency and severity, when the treatment is prolonged [7]. Other side effects of IFN-*β* (include injection site reactions, depression, liver injury, and hematologic abnormalities). However, so far, no effect on the vascular tone of the cerebral vessels has been reported, with the exception of a single case of reversible cerebral vasoconstriction syndrome (RCVS) described by Strohm et al. [8]. RCVS is generally associated with unusual headache episodes, with sudden onset and high intensity “thunderclap headache,” due to a widespread segmental vasoconstriction of the intracranial arteries, usually regressing within a three-month period. RCVS is probably triggered by the acute and transient alteration of the intracranial vascular tone regulation and usually presents a favourable development. Nonetheless, serious complications such as cerebral infarction, intracranial haemorrhage, or cerebral oedema may also occur. RCVS may be idiopathic or often secondary to the postpartum period or following the use of toxic agents or vasoactive drugs [9].

The contraction strength of the intracranial vessels, both intra- and extraparenchymal ones, can usually be evaluated through an angiography or through an angiomagnetic resonance imaging (Angio-MRI); however, transcranial Doppler (TCD) ultrasounds are more rapid and less invasive [10]. In fact, other earlier reported studies have demonstrated an inversely proportional relationship between the mean blood flow velocity (MFV) rate measured by TCD and the vessel diameter measured by means of angiography [10].

In our study we attempted to observe the intracranial haemodynamic changes in response to the IFN-*β* administration in patients with RRMS (primary end-point), by using TCD.

In addition, we investigated the possible differences between symptomatic and asymptomatic headache patients (secondary end-point) concerning cerebral haemodynamic.

## 2. Materials and Methods

We enrolled 20 MS-RR patients treated with a once week intramuscular administration of IFN-*β* 1A (Avonex®) consecutively examined and followed by the MS centre of the* “IRCCS Centro Neurolesi-Bonino Pulejo”* of Messina (Italy) from January 2016 to December 2016. The inclusion criteria were age range between 20 and 40 years, no drugs, and/or other administered substances (including tobacco smoke) that could affect the regulation of the vascular tone; absence of comorbidities that could potentially induce secondary headache; no history of headache prior initiation of interferon; conclusive diagnosis of RRMS according to Polman et al. criteria [11]; stable treatment with IFN-*β* 1A started by at least 6 months earlier.

We enrolled MS patients treated with the same formulation of IFN-*β* 1A in order to standardize the timing of performance of the TCD examination.

Avonex exerts its biological effects by binding to specific receptors on the surface of human cells.

This interaction gives rise to a complex cascade of intracellular events that lead to the expression of several interferon-induced gene products and markers, that is, MHC (Major Histocompatibility Complex) Class I, Mx protein, 2′/5′-oligoadenylate synthetase, P2 microglobulin, and neopterin. Some of these products were detected in the serum and in cellular fractions of the blood collected from patients treated with Avonex [5, 6, 12].

The pharmacokinetic profile of Avonex was then assessed using indirect method, by measuring its antiviral activity. Following intramuscular administration, serum antiviral activity levels reach their peak between 5 and 15 hours (generally an average of 10 hours) and gradually decrease down to the minimum activity, ascertained between 4th and 7th day after its administration [12].

During the screening visit, we collected all data concerning other potential side effects related to the administration of Avonex; in particular, previous episodes of headache before the beginning of treatment with IFN-*β* were inquired.

All the enrolled patients underwent a TCD immediately after Avonex administration (T0) and after 10 hours (T1), in accordance with previous pharmacokinetic studies.

The ultrasound protocol, performed with Motion-Mode module and according to standard technique [13], provided, through the temporal acoustic window, the analysis of the following arterial vessels: internal carotid artery (ICA) in its distal portion, middle cerebral artery (MCA), section A1 of the anterior cerebral artery (A1-ACA), and posterior cerebral artery (PCA), in order to assess the possible MFV rate variations in response to the administration of IFN-*β*.

At T0 and T1 heart rate (HR), systolic and diastolic blood pressure (SBP and DBP) of our patients were also recorded.

Twenty healthy volunteers (8 males and 12 females, between the ages of 18 and 40 years), after having undergone an anamnestic and a clinical evaluation to exclude headache and potentially inducing headache illness, were enrolled to obtain a normal range of TCD values.

### 2.1. Statistical Analysis

The analysis was conducted with descriptive statistic of respondent's demographic characteristic. Normality test of Shapiro-Wilks and Levene's variance homogeneity test were applied to the data. The data were not normally distributed and there was no homogeneity of variance between the groups. Sample clinical parameters were compared at T0 and T1 by the Wilcoxon signed-rank test in the MS group. The Mann–Whitney* U* test was used to compare differences between MS and healthy volunteers (HV) group. Correlations between demographic-clinical characteristic (age, disease duration, and initiation of therapy) and TCD parameters were computed by Spearman's coefficient. Subsequently, the sample was divided into two groups: headache and no-headache patients. The Wilcoxon signed-rank test was used in order to compare, for each group, the results between T0 and T1 (intragroups analysis), whereas the comparison between the two groups (intergroup analysis), at T0 and T1, was performed by mean of the Mann–Whitney* U* test. For intragroup analysis, Spearman's coefficient was used for the correlation. We performed a multiple regression analysis on the TCD parameters (dependent variables). At first, we focused on the influence of demographic and clinical variables, by using patient's age, disease duration, and initiation of therapy as predictors. We applied a backward stepwise elimination procedure for the choice of the best predictive variables according to the Akaike information criterion (AIC). Analyses were performed using an open source R3.0 software package. A 95% of confidence level was set with a 5% alpha error. Statistical significance was set at *p* < 0.05.

## 3. Results

### 3.1. Descriptive Analysis of Sample

Demographic and clinical features of our MS cohort are shown in Table 1.

In the entire MS cohort (headache + no-headache patients), we found a significant increase in at T1 in MFV-ICA (*p* = 0.03), MFV-MCA (*p* = 0.02), MFV-A1 ACA (*p* = 0.004), and MFV-PCA (*p* = 0.004). No significant correlations were found between disease duration, therapy duration, and clinical parameters. Intergroup analysis did not show significant difference between MS and HV group.

### 3.2. Intra-Inter Group Analysis

In intragroup analysis, we compared the clinical parameters at baseline (T0) and after IFN-*β* administration (T1) for each group (headache and no-headache patients). In headache group, we observed a significant increase in MFV-ICA (*p* = 0.05), MFV-MCA (*p* = 0.04), MFV-A1 ACA (*p* = 0.02), and MFV-PCA (*p* = 0.02). In the no-headache group, we did not find significant difference at T1. Intergroups analysis showed a significant trend between two groups at T0 in MFV-ICA (*p* = 0.06) (Table 2).

### 3.3. Correlation Analysis

In the headache group, we found many significant correlations between age, disease, and therapy duration with hemodynamic parameters (Table 3). In particular, age was positively correlated with MFV-ICA (*r* = 0.74; *p* = 0.03). In relation to disease duration, we observed a positive correlation with MFV-MCA (*r* = 0.71; *p* = 0.05). Concerning initiation of therapy, we found a trend with MFV-MCA (*r* = 0.67; *p* = 0.07). In the no-headache group, MFV-MCA was negatively correlated with disease duration (*r* = −0.68; *p* = 0.01) and therapy duration (*r* = −0.67; *p* = 0.02).

### 3.4. Multiple Regression Analysis

We found that the demographic and clinical features of the MS patients do not have a significant impact on TCD parameters (i.e., a significant trend was only found in relation to age influence in MFV-ICA and in disease and therapy duration influence on MFV-A1 ACA and MFV-PCA), as shown in Table 4.

## 4. Discussion

In our sample, MS patients showed a statistically significant difference in MFV rate (probably due to a vascular tone modification) in cerebral vessels in response to IFN-*β* administration. In particular, in the headache group, we observed a significant increase in MFV-ICA, MFV-MCA, MFV-A1 ACA, and MFV-PCA at T1. No significant correlations were found between disease duration, therapy duration, and haemodynamic parameters.

RCVS is a rare and often misdiagnosed syndrome whose pathophysiological causes are still unclear [9, 10] and, so far, only a single case associated with IFN-*β* 1A has been reported [8]. In this report, a twenty-year-old woman with recent MS diagnosis, following a two-month treatment with IFN-*β* 1A, developed a RCVS which regressed immediately after the immunomodulating drug was discontinued.

IFN-*β* 1A is a polypeptide normally produced by fibroblasts that ultimately induces antiproliferative and antiviral effects [5, 6]. Recognized side effects include flu-like symptoms, injection site reactions, severe cutaneous reaction, depression, liver injury, hematologic abnormalities, and headache [7]. Other reported side effects, involving vascular system, include Raynaud Phenomenon (RP), livedo reticularis (LR), and pulmonary artery hypertension [14, 15].

Particularly, Hanaoka et al. demonstrated that IFN-alpha (IFN-*β* pharmacologically related compound, with an expected similar side effect profile) induces pulmonary artery hypertension linked to a thromboxane cascade, with consequent elevated concentrations of thromboxane-B2 in plasma [16]. Currently, there are no experimental data related to the influence of IFN-*β* on activation of thromboxane cascade, but biochemical similarity may suggest a similar mechanism of action. Both the IFNs, in fact, induce expression of various cytokines [17] and increased plasma levels of IL-1, IL-2, IL-6, TNF alpha and interferon gamma were also observed in MS patients after IFN-*β* injection [17, 18]. Therefore, we hypothesize that some of these cytokines could induce cerebral vasoconstriction directly or through the activation of the thromboxane cascade.

### 4.1. Study Limitations

The relatively small sample size of the study groups and the lack of a control group for the effect of time (i.e., a group of MS patients that undergoes two testing sessions but does not take the drug) are the main limitations of the study.

## 5. Conclusion

Our data, together with literature findings, suggest that IFN-*β* could have vasoactive effects manifesting in multiple organ systems, probably mediated by a similar mechanism of IFN-alpha. Thus, we believe that clinicians should be aware of the vascular side effects of IFN-*β* and perform TDC in those MS patients developing headache during the immunomodulant treatment.

However, this work can be considered a pilot study and further efforts combining laboratory (cytokines dosage) and instrumental methods (TCD and Angio-MRI) are required to confirm these results.

## Figures and Tables

**Table 1:** Demographic and clinical characteristics of multiple sclerosis (MS) sample, frequencies (%).

	All subjects	Headache	No-headache
MS patients	20 (9 males, 11 females)	8 (3 males, 5 females)	12 (6 males, 6 females)
Age (years)	31.35 ± 5.56	31.62 ± 5.80	31.17 ± 5.64
Diseases duration (months)	61.3 ± 62.39	58.75 ± 63.61	63.0 ± 64.34
Therapy duration (months)	56.5 ± 59.01	55.25 ± 61.64	57.33 ± 59.96
Flu-like syndrome			
Yes	6 (30)	3 (37.5)	3 (25)
No	14 (70)	5 (62.5)	9 (75)

**Table 2:** Inter-group differences between healthy volunteers (HV) and multiple sclerosis (MS) group at baseline.

	HV group	MS group at Baseline	*p* value
	Median (first-third quartile)	Median (first-third quartile)	(*U* Mann–Whitney)
HR	79 (75.5–85.0)	80 (77.5–82.0)	0.97
SBP	120 (110.0–125.0)	117.5 (110.0–121.25)	0.42
DBP	77.5 (73.75–80.0)	80 (73.75–80.0)	0.79
MFV-ICA	50 (47.5–52.25)	50 (48.0–51.0)	0.76
MFV- MCA	60 (58.0–62.0)	59.5 (58.0–62.0)	0.98
MFV-A1 ACA	45 (43.0–45.25)	45 (42.0–45.0)	0.71
MFV-PCA	40.5 (38.75–42.0)	40 (38.0–40.25)	0.48

HR: heart ratio, SBP: systolic blood pressure; DBP: diastolic blood pressure; MFV-ICA: mean blood flow velocity internal carotid artery; MFV-MCA: mean blood flow velocity middle cerebral artery; MFV-A1 ACA: mean blood flow velocity section A1 of the anterior cerebral artery; MFV-PCA: mean blood flow velocity posterior cerebral artery.

**Table 3:** Intra- and intergroup differences at T0-T1 in headache and no-headache multiple sclerosis (MS) groups.

	Headache MS group Median (first-third quartile)	No-headache MS group Median (first-third quartile)	*p* value (U Mann–Whitney)
HR T0	81 (76–88.5)	77 (72.5–82.5)	0.26
HR T1	83 (77.5–90)	77 (73.5–78.5)	0.10
*p* value (Wilcoxon)	0.62	1.00	

SBP T0	122.5 (115–130)	117.5 (110–125)	0.37
SBP T1	120 (117.5–125)	115 (110–121.25)	0.31
*p* value (Wilcoxon)	1.00	0.52	

DBP T0	80 (75–81.25)	75 (70–80)	0.17
DBP T1	80 (78.75–81.25)	72.5 (70–80)	0.07
*p* value (Wilcoxon)	1.00	0.42	

MFV-ICA T0	47 (45.5–49.75)	51 (49.75–53.5)	0.06
MFV-ICA T1	51 (48–53)	52.5 (48–53.25)	0.58
*p* value (Wilcoxon)	0.05^*∗*^	0.44	

MFV-MCA T0	58 (58–60.25)	60 (58.75–62.25)	0.29
MFV-MCA T1	63 (59.75–68)	60 (59–62.25)	0.27
*p* value (Wilcoxon)	0.04^*∗*^	0.38	

MFV-A1 ACA T0	45 (44.5–45.25)	45 (42.5–45.25)	0.60
MFV-A1 ACA T1	47 (45.5–50.25)	45 (44–46)	0.15
*p* value (Wilcoxon)	0.02^*∗*^	0.10	

MFV-PCA T0	40.5 (38.75–41.25)	40.5 (38.75–42)	0.78
MFV-PCA T1	42 (40–44.25)	41 (40–42.25)	0.38
*p* value (Wilcoxon)	0.02^*∗*^	0.12	

HR: heart ratio, SBP: systolic blood pressure; DBP: diastolic blood pressure; MFV-ICA: mean blood flow velocity internal carotid artery; MFV-MCA: mean blood flow velocity middle cerebral artery; MFV-A1 ACA: mean blood flow velocity section A1 of the anterior cerebral artery; MFV-PCA: mean blood flow velocity posterior cerebral artery.  ^*∗*^*p* < 0.05.

**Table 4:** Backward linear regression: significant predictors on the TCD parameters.

Dependent variables	Predictors	*β*	Std *β*	*p* value	Adjusted *R*^2^
MFV-ICA	Age	0.43	0.65	0.06	0.06

MFV-A1 ACA	Disease duration	0.23	6.11	0.08	0.21
	Therapy duration	−0.24	−6.27	0.07	

MFV-PCA	Disease duration	0.20	6.85	0.07	0.15
	Therapy duration	−0.21	−6.77	0.06	

*β*: regression coefficient; Std *β*: standardized regression coefficient; MFV-ICA: mean blood flow velocity internal carotid artery; MFV-A1 ACA: mean blood flow velocity section A1 of the anterior cerebral artery; MFV-PCA: mean blood flow velocity posterior cerebral artery.
